# Two new species and new record of *Batricavus* Yin & Li (Coleoptera, Staphylinidae, Pselaphinae) from China

**DOI:** 10.3897/zookeys.215.3570

**Published:** 2012-08-17

**Authors:** Zi-Wei Yin, Li-Zhen Li

**Affiliations:** 1 Department of Biology, College of Life and Environmental Sciences, Shanghai Normal University, Shanghai, 200234, P. R. China

**Keywords:** Coleoptera, Staphylinidae, Pselaphinae, *Batricavus*, new species, new record, China, key

## Abstract

Two new species, *Batricavus abdominalis* Yin & Li, **sp. n.** and *Batricavus hainanensis* Yin & Li, **sp. n.** are described from Hainan, South China, with male habitus and major diagnostic features illustrated. *Batricavus tibialis* Yin & Li is newly recorded from Zhejiang, East China. Keys to both sexes of the genus are included.

## Introduction

The monotypic genus *Batricavus* Yin & Li presently contains only one species, *Batricavus tibialis* Yin & Li from Guangdong, South China. The genus is separated from the allies primarily by the presence of a pair of anteropronotal foveiform pits in both sexes (for further discussion see Yin et al. 2011: 530). Recently, a series of *Batricavus*, representing two undescribed species, was collected during an expedition to Hainan Island. In additional, two malespecimens representing a new record of *Batricavus tibialis*, collected from South Zhejiang by Jiang-Qing Zhu, became available for study. This paper will describe the new species, provide new distribution data for known species, and present identification keys to both males and females of the genus.

## Material and methods

Material treated in this study are housed in the Insect Collection of Shanghai Normal University, Shanghai, China (**SNUC**).

A slash (/) is used to separate lines on the same label, a double slash (//) is used to separate different labels. The terminology of the foveal system follows Chandler 2001, except for use ‘ventrite’ instead of ‘sternite’ when concerning the meso- and metathoracic structures.

The following acronyms are used in the text: **BL** – length of the body (= HL+PL+EL+AL); **HL** – length of the head from the anterior margin of the clypeus to the occipital constriction; **HW** – maximum width of the head across eyes; **PL** – length of the pronotum along the midline; **PW** – maximum with of the pronotum; **EL** – length of the elytra along the suture; **EW** – maximum width of the elytra; **AL** – length of the abdomen along the midline; **AW** – maximum width of the abdomen.

## Taxonomy

### Key to males

**Table:** 

1	Protibiae expanded laterally at apical 1/4 (Yin et al. 2011: 4, fig. 8) or near middle (Fig. 3A); metafemora and tergite IV simple	2
–	Protibiae simple; metafemora broadened mesally at apical 1/4 (Fig. 2B); tergite IV with large median cavity (Fig. 2A). (Hainan)	*Batricavus abdominalis* sp. n.
2	Antennomeres VIII much larger than VII (Yin et al. 2011: 4, fig. 6); protibiae expanded laterally at apical 1/4. (Guangdong, Zhejiang)	*Batricavus tibialis* Yin & Li
–	Antennomeres VIII smaller than VII (Fig. 1B); protibiae expanded laterally near middle. (Hainan)	*Batricavus hainanensis* sp. n.

### Key to females

**Table:** 

1	Elytra and abdomen scattered with long setae except for normal pubescence	*Batricavus abdominalis* sp. n.
–	Elytra and abdomen normally pubescent, lacking long setae	2
2	Head with basomedian carina extending anteriorly to level of posterior margin of vertexal foveae; tergite VIII with smooth posterior margin (Yin et al. 2011: 533, fig. 11)	*Batricavus tibialis* Yin & Li
–	Head with basomedian carina extending anteriorly for very short distance, followed by short, thin sulcus reaching anteriorly and ending as small round pit at level of posterior margin of vertexal foveae; tergite VIII with serrate posterior margin (Figs 3D, E)	*Batricavus hainanensis* sp. n.

#### Batricavus abdominalis

Yin & Li sp. n.

urn:lsid:zoobank.org:act:174178C3-F08F-4BD5-B9E9-EF2FAA2F120B

http://species-id.net/wiki/Batricavus_abdominalis

Figs 1A 2

##### Type-locality.

China, Hainan Province: Lingshui County, Diaoluoshan Natural Reserve, 18°43'36.93"N, 109°52'55.21"E, 950 m a.s.l.

##### Type material.

(8 ♂♂, 24 ♀♀) Holotype: ♂, labeled, ‘China: Hainan Prov. / Diaoluoshan N. R. / road to reservoir / 25.iv.2012, 950 m / Pan et Li leg.’. Paratypes: 2 ♂, 8 ♀♀, same label data as holotype; 1♂, 1♀, same label data except ‘Zi-Wei Yin leg.’; 4 ♂♂, 15 ♀♀, same label data except ‘Dai et Peng leg.’.

##### Other material examined.

5 ♂♂, 6 ♀♀, labeled ‘China: Hainan Prov. / Ledong County / Jianfengling N. R. / 15.xi.2005 / Jian-Hua Huang leg.’.

##### Diagnosis.

Length 1.50–1.60; antennal club three-segmented, lacking obvious modification in male; male with simple protibiae and modified metafemora and tergite IV.

##### Description.

**Figure 1.**
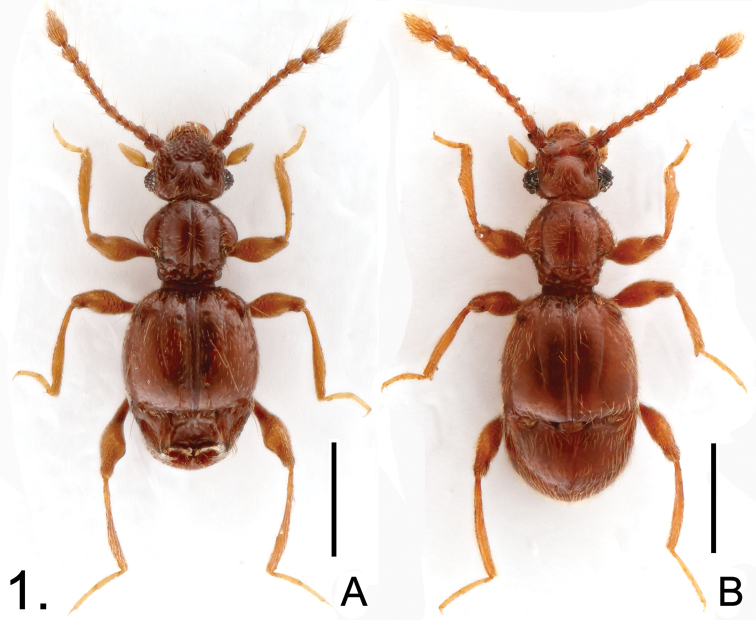
Male habitus of *Batricavus* **A** *Batricavus abdominalis* **B** *Batricavus hainanensis*. Scales: 0.5 mm.

**Figure 2.**
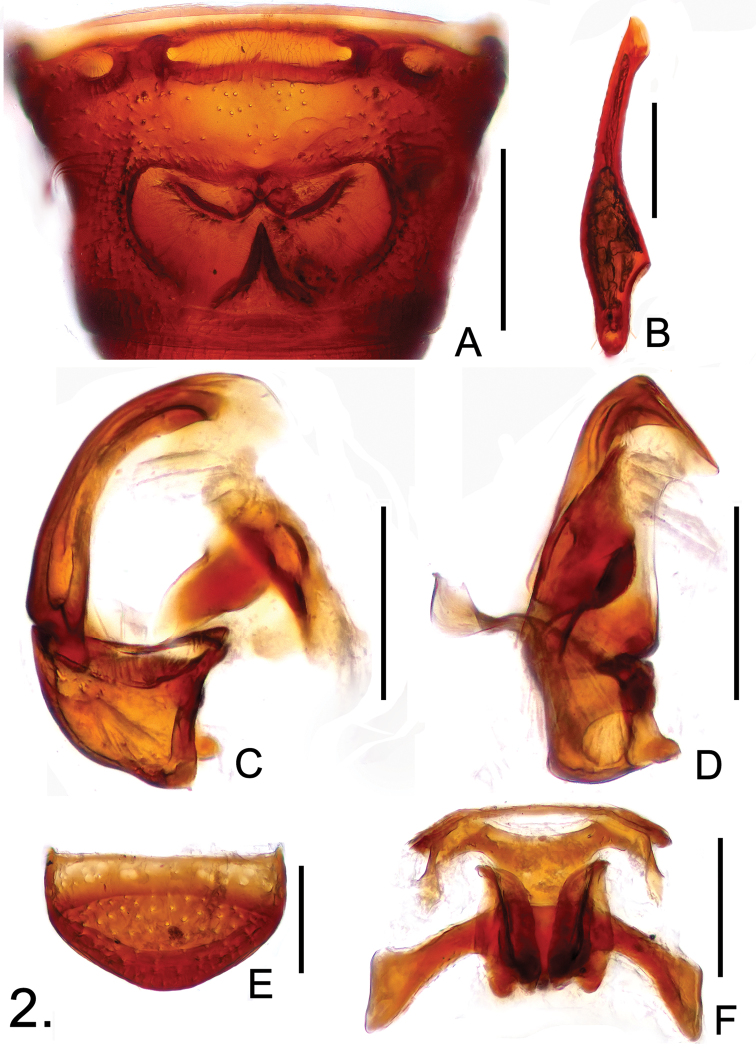
Diagnostic features of *Batricavus abdominalis* **A** male tergite IV **B** male metafemur **C** aedeagus, in lateral view **D** same, in ventral view **E** female tergite VIII **F** female gential complex, in ventral view. Scales: A, B = 0.2 mm, C–F = 0.1 mm.

Male. Length 1.50–1.59; body (Fig. 1A) reddish brown, maxillary palpi and tarsi lighter. Head slightly wider than long, HL 0.34–0.35, HW 0.39–0.41. Eyes each composed of about 20 large facets. Antennal club three-segmented, segment VIII smallest, IX–XI enlarged. Pronotum rounded laterally, slightly wider than long, PL 0.38–0.40, PW 0.42–0.46. Elytra wider than long, EL 0.53–0.57, EW 0.61–0.63, with scattered long setae. Protibiae simple; metafemora broadened mesally at apical 1/4 (Fig. 2B). Abdomen much narrower than elytra, AL 0.25–0.27, AW 0.53–0.55; tergite IV (Fig. 2A) with large median cavity comprising complicated structure. Aedeagus (Figs 2C, D) length 0.20, well-sclerotized, with elongate dorsal lobe.

Female. Measurements: BL 1.54–1.60, HL 0.33–0.34, HW 0.40–0.41, PL 0.37–0.38, PW 0.41–0.43, EL 0.50–0.53, EW 0.60–0.63, AL 0.34–0.35, AW 0.56–0.58.

Generally similar to male; eyes smaller, each composed of about 15 facets; tergite VIII (Fig. 2E) with smooth posterior margin; genital complex (Fig. 2F) transverse, width 0.24, well sclerotized.

##### Distribution.

South China: Hainan.

##### Etymology.

The specific epithet refers to the male sexual character present on abdominal tergite IV.

#### Batricavus hainanensis

Yin & Li sp. n.

urn:lsid:zoobank.org:act:B16612EB-1867-40DA-9298-6A001C992718

http://species-id.net/wiki/Batricavus_hainanensis

Figs 1B 3

##### Type-locality.

China, Hainan Province: Lingshui County, Diaoluoshan Natural Reserve, 18°42'18.87" N, 109°52'59.48"E, 720 m a.s.l.

##### Type material.

(2 ♂♂, 5 ♀♀)Holotype: ♂, labeled, ‘China: Hainan Prov. / Diaoluoshan N. R. / disc road / 26.iv.2012, 600–1,000 m / Dai et Peng leg.’. Paratypes: 1 ♂, 2 ♀♀, same label data as holotype; 1 ♂, same label data except ‘road to reservoir / 25.iv.2012, 1,000 m’; 2 ♀♀, same label data except ‘Zi-Wei Yin leg’.

##### Other material examined.

1 ♀, labeled ‘China: Hainan Prov. / Jianfengling N. R. / Wufenqu area / 2.v.2012, alt. 900 m / Pan et Yin leg.’.

##### Diagnosis.

Length 1.62–1.69; antennal club weakly indicated by apical three antennomeres, lacking obvious modification in male; male with modified protibiae and simple metafemora and tergite IV.

##### Description.

**Figure 3.**
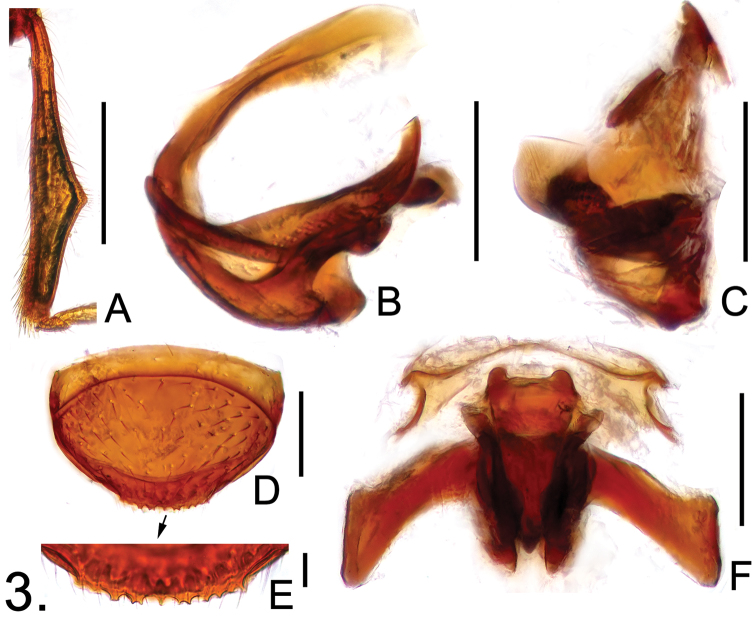
Diagnostic features of *Batricavus hainanensis* **A** male protibia **B** aedeagus, in lateral view **C** same, in ventral view **D** female tergite VIII **E** same, posterior margin enlarged **F** female gential complex, in ventral view. Scales: A = 0.2 mm, B–D, F = 0.1 mm, E = 0.02 mm.

Male. Length 1.62–1.67; body (Fig. 1B) reddish brown, maxillary palpi and tarsi lighter. Head slightly wider than long, HL 0.32–0.33, HW 0.39–0.41. Eyes each composed of about 30 facets. Antennal club formed by apical three antennomeres, segment VIII smallest, IX–XI enlarged. Pronotum rounded laterally, about as long as wide, PL 0.39–0.40, PW 0.40–0.41. Elytra wider than long, EL 0.56–0.58, EW 0.63–0.64, covered by short pubescence. Protibiae (Fig. 3A) triangularly expanded laterally near middle; metafemora simple. Abdomen narrower than elytra, AL 0.35–0.36, AW 0.58–0.59; tergite IV simple in structure. Aedeagus (Figs 3B, C) length 0.18, well-sclerotized, with elongate dorsal lobe.

Female. Measurements: BL 1.65–1.69, HL 0.34–0.35, HW 0.40–0.41, PL 0.38–0.39, PW 0.40–0.41, EL 0.53–0.54, EW 0.62–0.63, AL 0.40–0.41, AW 0.58–0.60.

Generally similar to male; eyes smaller, each composed of about 25 facets; tergite VIII (Fig. 3D, E) with serrate posterior margin; genital complex (Fig. 3F) transverse, width 0.27, well sclerotized.

##### Distribution.

South China: Hainan.

##### Etymology.

The specific epithet refers to the type locality.

#### Batricavus tibialis

Yin & Li, 2011

http://species-id.net/wiki/Batricavus_tibialis

Batricavus tibialis Yin & Li, 2011: 532.

##### Material examined.

2 ♂♂, labeled ‘China: Zhejiang Prov. / Jingqing County / Bai-Yun forest area / 7.v.2012, 1,200 m / Jian-Qing Zhu leg.’.

##### Distribution.

South China: Guangdong; East China: Zhejiang (new provinc record).

##### Remarks.

The two male specimens from Zhejiang are readily identified as *Batricavus tibialis* because they have the male sexual characters (present on antennae and protibiae) and the aedeagal form perfectly agrees with those of the type series from Guangdong.

## Supplementary Material

XML Treatment for Batricavus abdominalis

XML Treatment for Batricavus hainanensis

XML Treatment for Batricavus tibialis
